# Geography of public service delivery in rural Ethiopia

**DOI:** 10.1016/j.worlddev.2020.105133

**Published:** 2020-12

**Authors:** Gashaw T. Abate, Mekdim Dereje, Kalle Hirvonen, Bart Minten

**Affiliations:** a Markets, Trade, and Institutions Division of the International Food Policy Research Institute (IFPRI), Ethiopia; b Center for Development Research (ZEF), University of Bonn, Germany; c Development Strategy and Governance Division of IFPRI, Ethiopia; d Development Strategy and Governance Division of IFPRI, Myanmar

**Keywords:** Extension services, Remoteness, Ethiopia, Africa

## Abstract

Remote areas are often characterized by lower welfare outcomes due to economic disadvantages and higher transaction costs for trade. But their poorer situation may also be linked to worse public service delivery. Relying on large household surveys in rural Ethiopia, we explore this by assessing the association of two measures of remoteness – (1) the distance of service centers to district capitals and (2) the distance of households to service centers (the last mile) – with public service delivery in agriculture and health sectors. In the agriculture sector, we document statistically significant and economically meaningful associations between exposure to agriculture extension and the two measures of remoteness. For health extension, only the last mile matters. These differences between the two sectors could be due to the fact that more remote villages tend to have fewer agriculture extension workers who also put in fewer hours than their peers in more connected areas. This does not apply in the health sector. These findings provide valuable inputs for policymakers aiming to improve inclusiveness in poor rural areas.

## Highlights

• Geography has been shown to be an important determinant of economic development.
• We study how geography shapes public service delivery in rural Ethiopia.
• Access to agriculture (but not health) extension is lower in more remote villages.
• More remote villages have fewer agricultural extension workers who also work less.
• Access to both services declines with household's distance to the service center.

## 1 Introduction

Geography has been shown to be an important determinant of economic development. For example, it has been argued that landlocked countries – which are predominant in Africa – have performed economically worse as isolation and distance from the coast or navigable waterways are important deterrents to trade and, therefore, a cause of their sluggish growth and higher poverty (Bloom et al., 1998, Collier and Gunning, 1999, Limao and Venables, 2001, Sachs et al., 2001).^1^ At the micro-level, detailed household surveys in different countries have shown how modern input prices are higher and adoption rates of improved technologies, agricultural yields, commercial surpluses, and both non-farm and overall incomes are lower in more isolated areas (Christiaensen et al., 2003, Damania et al., 2017, Deichmann et al., 2009, Jacoby and Minten, 2009, Jacoby, 2000, Stifel et al., 2016). There is also compelling evidence of the existence of geographic poverty traps, according to which the geographic characteristics of the residence alone can lock people into poverty (Kraay & McKenzie, 2014). These insights have led a number of anti-poverty interventions to use geographical targeting to achieve their objectives (Baker and Grosh, 1994, Bigman and Fofack, 2000).

A number of authors have looked at specific economic sectors in analyzing links with geography. Substantial work has been done on the agricultural sector, building on the seminal work of von Thünen (1826), who showed strong differential agricultural patterns by distance to cities. Krugman (1991) argued how the interaction of transportation costs with differential economies of scale (increasing for manufacturing and constant for agriculture) leads to an often-observed industrial core and agricultural periphery development pattern. Gollin and Rogerson (2014) find that reductions in transportation costs could increase agricultural productivity and lead to a sizable re-allocation of labor into more productive activities in Uganda. The large impact of market access on agricultural practices has further been confirmed by specifically designed studies in different countries in Africa (Stifel and Minten, 2008, Vandercasteelen et al., 2018). Finally, in Ethiopia, recent evidence suggest that more connected areas are less vulnerable to weather shocks (Hill and Fuje, 2016, Hirvonen et al., 2020, Nakamura et al., 2020).

Strong geographical patterns emerge also in social sectors, such as education and health. For example, Alesina, Hohmann, Michalopoulos, and Papaioannou (2019) recently showed how intergenerational mobility in educational attainment in Africa is strongly shaped by transportation networks constructed by colonial authorities and by location – educational attainment mobility is higher close to the coast, near the capital, and in areas without malaria. For nutrition, dietary quality and chronic undernutrition among young children have been shown to vary along access to markets and other remoteness measures (Headey et al., 2018, Hirvonen and Hoddinott, 2017, Hirvonen et al., 2017, Stifel and Minten, 2017).

Perhaps a less explored reason for these worse outcomes relates to limited service delivery in more remote localities. Using data from 21,000 interviews in 17 African countries, Brinkerhoff, Wetterberg, and Wibbels (2018) show how remoteness is strongly related to access to and satisfaction with public services in rural Africa. Briggs (2018) finds that less connected areas in Africa are less likely to receive food aid. Fafchamps and Moser (2003) reported higher crime and insecurity in isolated areas of Madagascar. In the health sector, more connected and wealthier localities tend to have better access to health care than more remote and poorer areas (Dussault & Franceschini, 2006; Das & Gertler, 2007). In curative and preventive care, quality has been found to decline in poor and more remote areas because physicians operating in these areas tend to be less competent and put in less effort than their counterparts in more connected areas (Das and Gertler, 2007, Das et al., 2008, Leonard and Masatu, 2007), which may lead households to seek care outside their nearest health care facility (Klemick et al., 2009, Kruk et al., 2009, Leonard et al., 2002).

We study how geography shapes public service delivery in rural Ethiopia and contribute to the literature in three ways. Our first contribution is to consider two types of remoteness. First, we assess how public service delivery differs between remote and less remote villages within the same administrative areas. Second, we study how the remoteness of households from the service center is associated with differential access in rural areas in Ethiopia. This distinction is important as it leads to different policy implications to ensure the inclusiveness of remote areas or households in service provision. Our second contribution is to contrast two important sectors – agriculture and health – in which public service provisions play a vital role for achieving good performance. Our third contribution is to explore how remoteness is associated with the overall contact with extension workers but also with specific services they provide, such as advice on fertilizers, seeds and crops or antenatal care. We also study how extension workers' effort levels (e.g., hours worked) and quality (e.g., work experience, education level and knowledge) vary between remote and less remote villages.

We rely on two large and geographically widespread household surveys to study access to public services in the agriculture and health sectors in rural Ethiopia. Ethiopia is an interesting case study for a number of reasons. First, the large majority of the 100 million Ethiopians live in rural areas and, while the country has invested heavily in the construction of roads in the last decade, remoteness is still a dominant characteristic of rural life. Schmidt, Dorosh, Jemal, and Smart (2018) estimated that in 2015 more than 20 percent of Ethiopians were residing more than five hours travel time from a city of at least 50,000 people. Second, Ethiopia has relied on a 'developmental state' model for the delivery of a large number of social and extension services of which the public sector is typically the sole provider. Finally, while innovative technological solutions to aid service delivery are beginning to emerge (Abate et al., 2019, Desta et al., 2014), both agriculture and health extension services continue to rely primarily on face-to-face interaction either at the village service center or at the clients' home or parcel (Berhane et al., 2018, Mangham-Jefferies et al., 2014).

Using multivariable and local polynomial regression methods, we find that exposure to agriculture extension decreases as we move from more connected villages to less connected villages. But relative remoteness within village, i.e., the last mile, also matters; households farther away from village centers are less likely to have contact with agriculture extension agents. In the health sector, the location of the village is not correlated with exposure to services, but the last mile matters; households and pregnant mothers located farther away from the health posts are considerably less likely to receive key health services. Findings from exploratory analysis using survey data with the extension workers suggests that these differences are driven by differences in person-hours:more remote villages tend to have fewer agriculture extension workers who also put in fewer working hours than their peers in more connected areas. But we do not document similar associations in the health sector. Finally, for both sectors we find suggestive evidence that the quality of the extension workers declines as we move to more remote areas; extension workers in remote communities are younger and are less experienced, educated, and knowledgeable.

## 2 Public service delivery in Ethiopia

Ethiopia has seen successful human and agricultural development in the last decade as measured by changes in a number of indicators in the social sectors and in agriculture. In the agricultural sector, increasing adoption of improved modern inputs, such as chemical fertilizer and improved seed, has led to considerable yield increases, estimated at approximately five percent annually over the last decade (Bachewe, Berhane, Minten, &, Taffesse, 2018a). In the health sector, indicators for maternal and child health, addressing communicable diseases, improving hygiene and sanitation, and enhancing knowledge and health care seeking improved significantly (CSA and ICF, 2016). Chronic under-nutrition (stunting) among young children decreased from 58 percent in 2000 to 38 percent in 2016 (Golan, Headey, Hirvonen, & Hoddinott, 2019). Success in both sectors has been partly explained by increased outreach by extension agents (e.g. Bachewe et al., 2018b, Assefa et al., 2019).

In agriculture, the government of Ethiopia has made sizable investments to set up a large-scale public agricultural extension system (Abate et al., 2019, Davis et al., 2010).^2^ The effort has focused on the provision of advisory and training services through a public extension structure that extends from the federal ministry to the regions and down to the woreda (district) and kebeles (sub-district) through frontline extension agents. In the beginning of the 2000s, training of Development Agents (DAs) was scaled up through the establishment of Agricultural Technical and Vocational Education and Training (ATVET) colleges throughout the country. The intent was to assign at least three DAs (specializing in crop production, livestock, and natural resources, respectively) in each kebele. The new DAs were trained and mandated to carry out agricultural extension services to train farmers in each kebele. Each kebele planned to build a Farmer Training Center (FTC) where farmers would have access to participatory demonstrations for improved technologies and new farming systems. By 2008 and 2009, the ATVET colleges had trained some 60,000 DAs, and around 8500 FTCs have been established in the kebeles (Davis et al., 2010). Relying on data from the Ministry of Agriculture, Berhane et al. (2018) report that more than 72,000 DAs served about 16.7 million smallholder farmers in 2016/17 – that is, one DA per 230 farmers or 43 DAs per 10,000 farmers, one of the highest extension agent-to-farmer ratios in the world.

In the health sector, a health extension program (HEP) was launched in 2002/03 by the Ethiopian government, and now covers nearly all woredas of the country.^3^ The program focuses on hygiene and environmental sanitation, disease prevention and control, and family health services. The health extension workers (HEWs) play a key role in implementing the health extension program. To this end, the HEP has trained and deployed over 42,000 HEWs to kebeles (Assefa et al., 2019). There is one health post per kebele, and typically two HEWs are assigned to each health post and are responsible for reaching approximately 5000 individuals (Lemma & Matji, 2013). They are usually women who speak the local language. HEWs are selected by a committee nominated by the kebele, appended with representatives from the woreda health and other offices (Wang, Tesfaye, Ramana, & Chekagn, 2016). Before deployment, HEWs receive theoretical training in training stations and hands-on training in health centers (Wang et al., 2016). Together, this training lasts approximately 12 months. Once deployed, the HEWs are expected to reside in the kebele. Their main tasks include the provision of basic health services and the promotion of health-related messages. The HEWs are also responsible for training and supervising the so-called Health Development Army, a group of local volunteers tasked to support the HEWs.

## 3 Data

We use two secondary data sets collected by the authors in 2017–2018. The original purpose of these surveys was to conduct an impact evaluation of an agriculture and health (nutrition) program in Ethiopia. Consequently, the sampling designs and the survey instruments are slightly different in each survey. We therefore describe both surveys separately.

### 3.1 Agriculture extension

To analyze service delivery in the agriculture sector, we use data from a rich household survey of 2422 farm households. The primary purpose for collecting these data was to evaluate the impact of Digital Green’s pilot on video-mediated extension in Ethiopia.^4^ The post-intervention survey^5^ used here was administered between January and March 2018. In terms of geography, the survey covered 30 woredas and 346 kebeles in the four main agricultural areas of the country, i.e., Amhara; Oromia; Southern Nations, Nationalities, and Peoples' (SNNP); and Tigray regions. Fig. A1 in the online appendix shows the locations of these woredas. Farm households were selected using a four-stage sampling process. First, 30 woredas were purposefully selected from the total of 68 Digital Green pilot woredas.^6^ Second, up to 15 kebeles were randomly selected from each sample woreda.^7^ Third, three development groups^8^ were randomly selected from each sample kebele.^9^ At this stage, the sample was further stratified by distance of the development groups to the kebele center to ensure that the sample represented both farm households that reside close to and far away from the kebele service point. In the last stage, seven farm households were randomly selected from each kebele, i.e. two from the closest development group, two from the farthest development group, and three from the development group situated at the median distance from the kebele center.

While this survey was not designed to represent the four regions, it has a number of useful characteristics for the purposes of this study. First, it is geographically widespread covering 30 woredas in four regions. Second, within each of the selected woredas, we have a sizable number of kebeles (9–15 kebeles) and farm households (63–105 households). Third, the household questionnaire contained extremely detailed questions about farmers' contact with the local DAs. Finally, the survey teams also interviewed the DAs operating in the same communities.

Table 1 shows descriptive statistics for the 2422 households in our sample. About 40 percent of the farmers reported that their plots have been visited by a DA during the 2017/18 main cropping (*meher)* season. About half of the households reported to have received plot level advice on crop choice and fertilizer and seed use from their local DAs.

**Table 1:** Descriptive statistics – cereal survey.

	Mean	Standard deviation	Min	Max
*Exposure to extension services:*				
DA visited the plot (0/1)	0.4	0.5	0	1
DA provided advice on amount of fertilizer to use on the plot (0/1)	0.5	0.5	0	1
DA provided advice on type of fertilizer to use on the plot (0/1)	0.5	0.5	0	1
DA provided advice on type of seed to use on the plot (0/1)	0.5	0.5	0	1
DA provided advice on the type of crop suitable to the plot (0/1)	0.5	0.5	0	1
DA provided crop specific advice on the plot (0/1)	0.6	0.5	0	1

*Distance variables:*				
Distance to the woreda capital by car (km)	19	12.2	1	61
Household's distance to FTC (minutes)	30.1	23.2	0	90

*Household characteristics:*				
Male head (0/1)	0.9	0.3	0	1
Age of the household head (years)	45.9	12.9	20	95
Education of household head (years of schooling)	3.3	3.8	0	17
Males 0–15 years of age (number)	1.4	1.2	0	7
Males 16–64 years of age (number)	1.6	1	0	10
Males 64+ years of age (number)	0.1	0.3	0	4
Females 0–15 years of age (number)	1.3	1.2	0	6
Females 16–64 years of age (number)	1.4	0.8	0	7
Females 64+ years of age (number)	0.1	0.2	0	2
Value of household assets (in '000 birr)	1.5	4.6	0	36.1

*Plot characteristics:*				
Land area cultivated (hectares)	3.4	3.5	0.1	18.1
Plots managed (number)	2.6	1.6	1	15
Slope of the plot is flat (0/1)	0.6	0.4	0	1
Soil is fertile (0/1)	0.3	0.4	0	1
Travel time to the plot from residence (minutes)	19.7	21.1	0	180

Source: Authors’ calculation based on the 2018 Digital Green's household survey data.

Note: N = 2422 farm households.

We used two measures of remoteness: distance between the kebele center and the woreda capital; and the distance of the household to the Farmers Training Center (FTC), which is typically located at the kebele center. The distance between the kebele and the woreda capital was estimated by the DA. The average kebele in our sample is located about 19 km away from the woreda capital. However, there is sizable variation in distances to the woreda center. While some kebeles are located just a kilometer away, residents of the most remote kebeles need to travel more than 60 km to reach the woreda center. For the last-mile distance measure, we asked sample households to estimate the average time required to reach the FTC using their usual means of transportation. As shown in Table 1, it takes 20 min for sample households to reach the FTC, on average. Again, for some households the distance is much longer with the most remote households traveling about one and half hours to reach the FTC. Fig. B1 in the online appendix provides full distributions for both distance variables.

The summary statistics on household characteristics indicate that the vast majority of sample households are male-headed (90 percent) with low education levels (3.3 years of schooling). The average household in our sample manages close to 3 plots with a total farm size of 3.4 ha, although only 30 percent of the plots are reportedly fertile. On average, the plots managed by the sample households are located just <20 min away from their dwelling.

The summary statistics based on the interviews with the 781 DAs are reported in Table 2. On average, there are more than three DAs per kebele, consistent with the government’s plan to assign at least three DAs per kebele, each specialized in either crop production, animal science, and natural resources management. A typical DA in our sample works about 36 h per week.

**Table 2:** Descriptive statistics – agricultural development agents (DAs).

	Mean	Standard deviation	Min	Max
Number of DAs in the kebele	3.5	1.3	1.0	8
Average working hours per week	36.0	14.5	3.0	84
Age (in completed years)	28.8	6.2	20.0	56
Education (diploma or higher)	0.8	0.4	0.0	1
Years of experience (in completed years)	6.7	5.1	0.1	26.5
Average technology test score	65.2	11.0	22.3	89.8

Source: Authors’ calculation based on Digital Green’s DA survey, 2018.

Note: 781 observations.

The average DA in our sample is 29 years old and has 7 years of work experience in agricultural extension. However, there is substantial variation both in age and years of experience. Eighty percent of the DAs in our sample held a college diploma or higher, indicating that DAs in Ethiopia are relatively highly educated.

We also measured the knowledge of DAs using a set of questions about selected technologies and practices that they promote. These questions were drawn directly from the technical extension guidelines drafted by the Ethiopian government. The knowledge tests were crop-specific, with 17 questions on teff, 16 on wheat, and 16 on maize – the main cereals that the agricultural extension service primarily focuses on. The questions were designed in multiple choice format, and we scored the responses to these questions on a 0 to 100 scale where 100 indicates that the respondent answered all questions correctly. As shown in Table 2, the average DA correctly answered about 65 percent of the questions across the three crops. Strikingly there are DAs who scored as low as 22 percent on their knowledge of technologies and practices they themselves promote.

### 3.2 Health extension

To assess the role of geography in health extension, we use data from a household survey administered in March 2017.^10^ The primary purpose of the survey was to serve as a baseline for an evaluation of nutrition-sensitive components of the fourth phase of the Productive Safety Net Program (PSNP).^11^ A stratified sample of 2,635 households with children <24 months of age was drawn from localities in which PSNP operates. In total, the survey covered 164 kebeles in 88 woredas in Amhara, Oromia, SNNP, and Tigray regions. Fig. A2 in the online appendix shows the locations of these woredas. The 88 woredas were randomly selected from the full list of PSNP woredas. Three kebeles were randomly selected from each woreda, and from each kebele, one enumeration area was randomly selected. After a full household listing of the enumeration area, 10 eligible households were randomly selected. The sample was further stratified so that roughly half of the selected households were PSNP beneficiaries and the other half were poor but not benefitting from the program.^12^ Given the focus on poor households with young children, this sample is not representative of the kebeles in which the sample was drawn.

While the geographic and demographic restrictions limit the representativeness of the sample, the survey has several useful characteristics. First, the sample is large and geographically widespread, covering 264 kebeles and 88 woredas in four regions. Second, although all 264 communities are poor, they vary substantially in terms of remoteness. Third, in addition to collecting data on households' access to health services, the survey teams visited and HEWs operating in these communities. Finally, we have GIS coordinates for both the surveyed households and the health posts, permitting us to compute distances between the two. After dropping 20 households with missing observations, we are left with 2615 households in our analytical data set.

Table 3 shows the summary statistics for the households in the final sample. In terms of exposure to the health extension workers, we see that 79 percent of the households know at least one HEW working in their kebele but only 36 percent of the households had contact with a HEW in the last 3 months. Meanwhile, 23 percent reported that a HEW had visited their home anytime, and 11 percent said this happened in the last 3 months. Seventy-five percent of mothers reported to have received antenatal care during their recent pregnancy, and 29 percent said that a HEW visited their home during the pregnancy.

**Table 3:** Descriptive statistics – health survey.

	Mean	Standard deviation	Min	Max
*Exposure to extension services:*				
Household knows a HEW working in the kebele	0.79	0.41	0	1
Household has met with HEW in the last 3 months	0.36	0.48	0	1
HEW ever visited household's home	0.23	0.42	0	1
HEW visited household's home in the last 3 months	0.11	0.31	0	1
HEW visited the mother during pregnancy	0.29	0.45	0	1
Mother received antenatal care during last pregnancy	0.75	0.44	0	1

*Distance variables:*				
Distance from community to woreda capital by car (min)	66.75	73.95	0	480
Distance from household to the health post (km)	2.49	3.12	0.02	41.35

*Household characteristics:*				
Youngest child is male	0.5	0.5	0	1
Youngest child's age in full months	11.44	6.75	0	29
Mother's age (years)	28.82	6.51	16	50
Mother's education (years of schooling)	0.87	2.24	0	13
Male head	0.89	0.31	0	1
Head's age (years)	38.01	10.8	17	100
Education of head (years of schooling)	1.1	2.56	0	13
Head is Orthodox	0.48	0.5	0	1
Head is Muslim	0.32	0.47	0	1
Males 0–5 years (number)	0.85	0.76	0	4
Males 6–15 years (number)	0.83	0.97	0	6
Males 16–60 years (number)	1.11	0.59	0	4
Males 61+ years (number)	0.05	0.22	0	2
Females 0–5 years (number)	0.89	0.76	0	4
Females 6–15 years (number)	0.82	0.98	0	6
Females 16–60 years (number)	1.18	0.47	1	6
Females 61+ years (number)	0.04	0.21	0	2
Land area operated (hectares)	0.87	0.81	0.0001	4.5
Number of tropical livestock units (TLU) owned	3.04	2.99	0	16.25

Source: Authors’ calculation based on 2017 Productive Safety Net Program (PSNP) survey data.

Note: N = 2615 households.

We asked the community leaders to estimate the average time required by car to reach the woreda capital. The responses ranged from zero to eight hours with the sample mean of just over one hour. We use the GIS coordinates to compute the distance between the health post and household's location. Fig. B2 in the online appendix provides full distributions for both distance variables. The average household resided 2.5 km from the health post. Some households resided in close proximity (20 m) while for others the distance was over 40 km.

The other summary statistics reported in Table 3 further indicate that these are typical rural Ethiopian households characterized by low education and asset levels.

Summary statistics based on the interviews with 249 HEWs are reported in Table 4.^13^ Consistent with the government guidelines, there are about 2 HEWs per kebele, on average. In 15 percent of the kebeles there was only one HEW, while in 23 percent of the kebeles there were more than two HEWs. The average HEW in our sample reported to have worked 212 h per month. Moreover, she is 27 years old and has 7 years of work experience as a HEW. The HEWs tend to be less educated than the DAs. About 3 percent of the HEWs reported to have only completed primary school, 8 percent secondary school, and 7 percent high school. More than 67 percent had completed technical or vocational training and 15 percent had a degree from a university or college.

**Table 4:** Descriptive statistics – health extension workers.

	Mean	Standard deviation	Min	Max
Number of HEWs in the kebele	2.14	0.73	1	5
Average working hours per month	212.7	53.3	40	420
Age, years	26.9	4.47	18	52
Years of experience	6.97	3.92	0.1	18

*Highest level of education:*				
Primary school	0.03	0.17	0	1
Secondary school	0.08	0.27	0	1
High school	0.07	0.26	0	1
Technical/vocational training	0.67	0.47	0	1
University/college diploma	0.15	0.36	0	1
Average IYCF test score	90.7	9.01	42.9	100

Source: Authors’ calculation based on the 2017 Productive Safety Net Program (PSNP) survey data.

Note: 249 observations.

One of the key tasks of the HEWs is the provision of nutrition counselling. We therefore tested HEWs knowledge about age-appropriate infant and young child feeding (IYCF) practices through a set of 14 multiple choice questions. The mean test score was 90 percent, indicating a very good understanding by the HEWs of recommended IYCF practices.

## 4 Econometric approach

Our econometric analysis focuses on the relationship between service delivery and remoteness in rural Ethiopia. We model the exposure to extension services of household *i* that resides in community *c* of woreda *w* ($E_{\mathrm{icw}}$) as a function of two measures of remoteness – the community's distance to the woreda capital ($F_{\mathrm{cw}}$) and the household's distance to the kebele center ($L_{\mathrm{icw}}$):

(1) $$E_{\mathrm{icw}}=\beta_{1}F_{\mathrm{cw}}+\beta_{2}L_{\mathrm{icw}}+X_{\mathrm{icw}}^{\text{'}}\delta +\omega_{w}+\varepsilon_{\mathrm{icw}},$$

where $X_{\mathrm{icw}}^{\text{'}}$ is vector of household characteristics and $\omega_{w}$ represents woreda-level fixed effects.^14^ The error term is captured by $\varepsilon_{\mathrm{icw}}$.

In the context of agriculture extension, we use six different binary exposure variables ($E_{\mathrm{icw}}$) capturing scenarios where the DA:

1. visited the household's plot in the previous agricultural (*meher* 2017–18) season;
2. provided advice on amount of fertilizer to use on the household's plot;
3. provided advice on type of fertilizer to use on the household's plot;
4. provided advice on type of seed to use on the plot;
5. provided advice on the type of crop suitable to the plot; and
6. provided crop specific advice on the plot.

To assess the exposure to health extension services, we use five binary outcome variables:

1. the household knows a HEW working in the kebele;
2. the household has met with a HEW in the last 3 months;
3. a HEW ever visited the household's home;
4. a HEW visited the mother during pregnancy; and
5. the mother received antenatal care during last pregnancy.

The use of several outcome variables raises a concern that some of the statistically significant *β* estimates are due to chance, i.e., false positives. To address this concern, we report p-values in our regression tables and then indicate if the p-value is below the Bonferroni-adjusted (B-adj.) critical value that accounts for the mean correlation across the outcome variables (Aker, Boumnijel, McClelland, & Tierney, 2016).

The survey instruments do not allow us to use uniform distance measures across the two surveys. In both surveys, the distance between the community and woreda capital ($F_{\mathrm{cw}}$) was estimated by the community leaders or the DA. In the agriculture survey, the distance from the community to the woreda capital is measured in terms of distance to the woreda capital by road in kilometers. In the case of health extension, the distance was measured as the travel time in minutes it takes by a car to reach the woreda center. We express this distance variable as a set of three binary variables that place kebeles located in the same kebele into terciles based on their distance to the woreda capital. The tercile capturing the kebeles closest to the woreda capital is selected as the reference group.

The way we measure the last mile ($L_{\mathrm{icw}}$) also varies across the surveys. In the agriculture extension survey, we asked the households to estimate the time in minutes it takes for them to walk to the FTC. In the health extension survey, we used GPS coordinates of the household and the health post and computed the distance in kilometers between the two points. In both cases, we express this last mile distance variable as a set of three binary variables that rank households into terciles based on the distance within the kebele. As before, the tercile capturing the households closest to the extension office is selected as the reference group.^15^

Households located in more remote woredas or more remote areas within the kebele may differ from less remote households in several ways that may also affect their demand for extension services. We control for many such factors by including household level controls to the estimated specification. These variables are listed in Table 1, Table 3. They vary somewhat between the two surveys to account for different factors that shape demand in the context of agriculture and health extension.

The woreda fixed effects $\omega_{w}$ capture all woreda level aspects, such as administrative capacity, climate, and infrastructure, which are shared by the households residing in the same woreda. While most of our dependent variables are binary ones, we use a linear probability model (instead of logit or probit) to better accommodate these woreda fixed effects. We account for the stratified sampling strategy in both surveys by clustering our standard errors at the woreda level.^16^

A common concern in observational studies such as ours is that the error term is correlated with the variables of interest. In our application, the two distance variables ($F_{\mathrm{cw}}$ and $L_{\mathrm{icw}}$) may well be correlated with the error term ($\varepsilon_{\mathrm{icw}}$). If so, our estimates for the *β*s would be biased. While we cannot rule this out, we note that our model addresses many sources of such correlation. In addition to controlling for woreda fixed effects, we control for several household characteristics that might be associated with remoteness and the demand or supply of extension services, e.g., education and wealth levels of the households. We also note that in Ethiopia, migrating to farm in less remote localities, be it outside or within the community, is difficult because of absent land markets. All land is owned by the state, and farmers only have usufruct rights to their land granted by local government authorities, who further privilege access to those who already live in that locality (Ambaye, 2012).

## 5 Results

### 5.1 Agricultural extension

Table 5 shows the regression results on exposure to agriculture extension. We document strong correlations between exposure to agriculture extension and advice along both distance gradients ($F_{\mathrm{cw}}$ and $L_{\mathrm{icw}}$) even after controlling for household characteristics and woreda fixed effects. The results on DAs visit (as measured by plot level visits) show that, relative to farm households in the least-remote kebeles, households located in the farthest kebeles had a 5 percentage points lower likelihood of a visit by a DA (column 1).^17^ Given that only 30 percent of our sample farmers were visited by the DA (Table 1), this translates into a 17 percent drop in the likelihood that a DA visited households in the most remote locations. The relative remoteness within one's kebele seems to matter even more. Farm households residing farthest from the FTC in their kebele had a 37 percent (11 percentage points) lower probability of a plot level visit by a DA (p < 0.01; B-adj. p < 0.1) compared to the least remote households. Households in the second tercile were also considerably less likely to receive these visits than the least remote households.

**Table 5:** Relationship between relative remoteness and exposure to agriculture extension.

	(1)	(2)	(3)	(4)	(5)	(6)
Outcome variable:	Was this plot visited by the Develop-ment Agent (DA)?	Did the DA advice on amount of fertilizer to use?	Did the DA advice on type of fertilizer to use?	Did the DA advice on type of seed to use on this plot?	Did a DA advice on the type of crop suitable to this plot?	Did the DA provide crop specific advice on the plot?
*Distance to woreda from kebele center (km):*						
First tercile	(reference)	(reference)	(reference)	(reference)	(reference)	(reference)
Second tercile	−0.024	−0.047	−0.051	−0.066*	−0.033	−0.000
	(0.032)	(0.035)	(0.034)	(0.035)	(0.030)	(0.032)
	[0.468]	[0.193]	[0.156]	[0.073]	[0.274]	[0.999]
Farthest tercile	−0.051*	−0.064*	−0.080**	−0.096***	−0.065**	−0.032
	(0.029)	(0.032)	(0.031)	(0.030)	(0.031)	(0.028)
	[0.099]	[0.052]^b^	[0.015]^b^	[0.004]^b^	[0.044]^b^	[0.261]

*Household's travel time to FTC (in minutes):*						
First tercile	(reference)	(reference)	(reference)	(reference)	(reference)	(reference)
Second tercile	−0.084***	−0.054**	−0.049*	−0.067**	−0.070**	−0.039
	(0.026)	(0.026)	(0.026)	(0.025)	(0.034)	(0.023)
	*[0.003] ^b^*	*[0.055]*	*[0.085]*	*[0.014] ^b^*	*[0.045] ^b^*	*[0.111]*
Farthest tercile	−0.109***	−0.074***	−0.067**	−0.075***	−0.075***	−0.048*
	(0.028)	(0.025)	(0.027)	(0.026)	(0.026)	(0.024)
	*[0.001]^b^*	*[0.006]^b^*	*[0.020]^b^*	*[0.008]^b^*	*[0.007]^b^*	*[0.055]*

Region dummies?	Yes	Yes	Yes	Yes	Yes	Yes
Household level controls?	Yes	Yes	Yes	Yes	Yes	Yes
Woreda fixed effects?	Yes	Yes	Yes	Yes	Yes	Yes

Observations	2367	2367	2367	2367	2367	2368
R^2^/within-R^2^	0.065	0.053	0.053	0.062	0.047	0.035
Adjusted R^2^/adjusted within-R^2^	0.058	0.045	0.045	0.054	0.039	0.027

Source: Authors’ calculation based on Digital Green’s DA survey, 2018.

Note: Standard errors clustered at the woreda level and reported in parentheses.

Statistical significance denoted by *p < 0.10, **p < 0.05, ***p < 0.01.

Bonferroni adjusted p-values in brackets and in italics. Bonferroni-adjusted critical value for 0.10 significance level, considering the average correlation between the six outcome variables, is 0.055. b indicates that the estimated p-value is below this threshold.

Remote households are also less likely to receive advice on fundamental inputs (fertilizer and seed) and crop specific practices. For instance, compared to households in more connected kebeles, farm households in most remote kebeles had 6 and 8 percentage points lower likelihood of receiving advice on the amount and type of fertilizer to use on their plots, respectively (columns 2 and 3). Similarly, remote households within the kebeles had a 7-percentage points (14 percent) lower probability of getting advice on fertilizer use than the least remote households (column 3). DA advice on the type of seed farmers should use in a plot is also limited in remote areas. Households in the most remote kebeles were 10 percentage point (or 23 percent) less likely to receive plot level advice on seed selection, while most remote households within their kebele had an 8 percentage point (or 18 percent) lower likelihood of receiving such advice (column 4). Households in the second tercile in terms of distance from the woreda or FTC were similarly disadvantaged relative to their peers in the least remote locations.

On crop specific advice, we find that farm households in distant kebeles were 7 percentage point less likely to receive advice on the type of crop suitable for their plots compared to households residing in kebeles closer to the woreda capital (column 5). Household's distance to the FTC is similarly associated with a decline in the likelihood of receiving this advice. Finally, we checked whether the access to crop specific DA advice varies by distance to service centers. While the distance of the kebele to woreda capital is not strongly correlated with the probability of receiving crop specific DA advice, the relative distance to the FTC is negatively associated with access to crop specific guidance, i.e., households in the most remote villages had a 5 percentage points (8 percent) less likelihood of receiving crop specific DA advice (column 6).^18^

These results also imply that *doubly* remote households, i.e., those located in remote kebeles and far away from the FTC in their kebele, are least likely to receive extension services. For example, adding up the coefficients reported in column 2 tell us that these *doubly* remote households are 14 percentage points (or 28 percent) less likely to receive advice on the amount of fertilizer to use on their plot than their counterparts located close to the FTC in least remote kebeles.^19^

### 5.2 Health extension

Table 6 reports the results with respect to health extension services. In contrast to agricultural extension, we see that household's exposure to health extension is not associated with distance to the woreda capital; all coefficients are small in magnitude and not statistically different from zero. However, we do document strong associations regarding exposure to health extension and household's distance to the health post, but mostly for households in the farthest tercile.

**Table 6:** Relationship between relative remoteness and exposure to health extension.

	(1)	(2)	(3)	(4)	(5)
Outcome variable:	Knows a Health Extension Worker (HEW) working in the kebele?	Has met with HEW in the last 3 months?	HEW ever visited home?	HEW visited during pregnancy?	Received antenatal care?
*Distance to woreda from kebele center (km):*					
First tercile	(reference)	(reference)	(reference)	(reference)	(reference)
Second tercile	−0.005	0.027	−0.001	−0.025	−0.012
	(0.027)	(0.023)	(0.023)	(0.025)	(0.024)
	*[0.853]*	*[0.233]*	*[0.973]*	*[0.305]*	*[0.630]*
Farthest tercile	−0.019	−0.004	−0.013	−0.019	−0.002
	(0.024)	(0.027)	(0.026)	(0.030)	(0.024)
	*[0.443]*	*[0.874]*	*[0.617]*	*[0.535]*	*[0.945]*

*Household's distance to health post (km):*					
First tercile	(reference)	(reference)	(reference)	(reference)	(reference)
Second tercile	−0.018	−0.048^**^	−0.025	−0.030	−0.012
	(0.018)	(0.022)	(0.020)	(0.019)	(0.019)
	*[0.319]*	*[0.034]*	*[0.209]*	*[0.126]*	*[0.507]*
Farthest tercile	−0.066***	−0.078***	−0.045**	−0.048**	−0.043**
	(0.019)	(0.022)	(0.020)	(0.019)	(0.019)
	*[0.001]^b^*	*[0.001]^b^*	*[0.025]^b^*	*[0.011]^b^*	*[0.030]^b^*

Region dummies?	Yes	Yes	Yes	Yes	Yes
Household level controls?	Yes	Yes	Yes	Yes	Yes
Woreda fixed effects?	Yes	Yes	Yes	Yes	Yes

Observations	2615	2615	2615	2615	2615
R^2^/within-R^2^	0.022	0.018	0.016	0.013	0.011
Adjusted R^2^/adjusted within-R^2^	0.013	0.009	0.008	0.005	0.002

Source: Authors’ calculation based on the 2017 Productive Safety Net Program (PSNP) survey data.

Note: Standard errors clustered at the woreda level and reported in parentheses.

Statistical significance denoted by *p < 0.10, **p < 0.05, ***p < 0.01.

Bonferroni adjusted p-values in brackets and in italics. Bonferroni-adjusted critical value for 0.10 significance level, considering the average correlation between the five outcome variables, is 0.032. b indicates that the estimated p-value is below this threshold.

Compared to least remote households, households located farthest away from the health post are 7 percentage points (p = 0.02; B-adj. p < 0.1) less likely to even know a health extension worker in their kebele (column 1). Considering that 79 percent of the households know at least one health extension worker operating in their kebele (see Table 3), this translates into a 11-percent lower likelihood of knowing a health extension worker between households in the first and last distance tercile.

The same story holds when we assess the likelihood of having met a health extension worker (columns 2 and 3). For example, health extension workers are less likely to visit homes that are located farther from the health post (column 3). Relative to the least remote households, households in the third distance tercile were 4.5 percentage points (p = 0.02; B-adj. p < 0.1) or 20 percent less likely to have been visited by a HEW (column 3).

Similar patterns emerge when we consider services to pregnant women. Pregnant mothers located farthest from the health post are 5 percentage points (p = 0.02; B-adj p < 0.1) less likely to receive a visit from a health extension worker as compared to those located close to the health post (column 4). Considering that 29 percent of the mothers reported to have been visited by a health extension worker during their pregnancy (see Table 3), this translates into a 17 percent fall in the likelihood of a visit when we move from the first distance tercile to the last one. Column 5 tells us that mothers residing far away from the health post are also less likely to receive antenatal care (p = 0.02; B-adj p < 0.1).

## 6 Remoteness and characteristics of public extension providers

The foregoing results put forward two interesting stylized facts. First, the last mile matters: more remote households within their kebeles are considerably less likely to receive both types of extension services; agriculture and health. Second, exposure to agriculture extension declines as we move from more connected kebeles to kebeles located far from the woreda capital. But interestingly, we do not document a negative gradient when it comes to exposure to health extension.

In this section, we explore some potential reasons for the difference between the two sectors. We do so by running a series of locally weighted polynomial regressions in which we regress various extension worker characteristics on the distance from the kebele to the woreda capital. We also replicated these results using regression models that control for woreda fixed effects. Tables E1 and E2 in the online appendix show the regression results based on the agriculture extension survey and on the health extension survey.

**Fig. 1:**
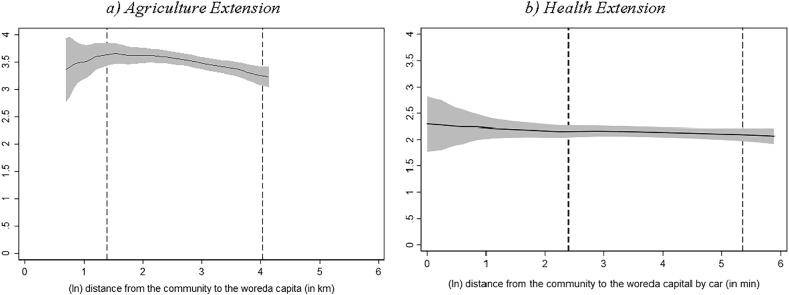
Number of extension agents in kebele and distance to woreda capital Source: Authors’ calculation based on Digital Green’s DA survey, 2018 and Productive Safety Net Program (PSNP) survey, 2017 Note: Local polynomial regression. The vertical axis in both graphs measures the number of extension workers operating in the kebele. The area between the dashed lines indicates the 90% of the distance distribution (between the kebele center and woreda capital). (For interpretation of the references to colour in this figure legend, the reader is referred to the web version of this article.)

**Fig. 2:**
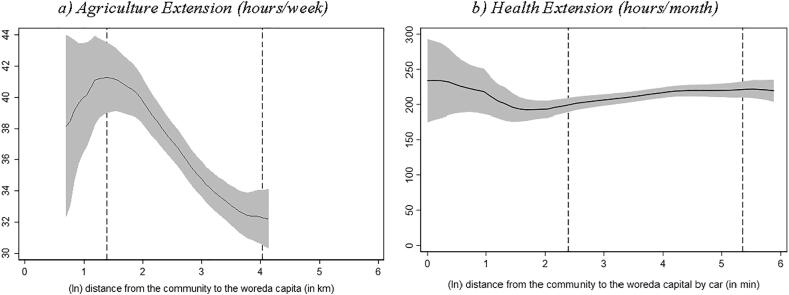
Reported working hours and distance to woreda capital Source: Authors’ calculation based on Digital Green’s DA survey, 2018 and Productive Safety Net Program (PSNP) survey, 2017. Note: Local polynomial regression. The vertical axis in graph a) measures the number of typical working hours per week reported by the agriculture extension worker. In graph b) the vertical axis measures the number of typical working hours per month reported by the health extension worker. The area between the dashed lines indicates the 90% of the distance distribution (between the kebele center and woreda capital). (For interpretation of the references to colour in this figure legend, the reader is referred to the web version of this article.)

Starting from Fig. 1, we see how the number of DAs declines as we move to more remote kebeles, but the same is not true for HEWs.^20^ Fig. 2 shows that DAs in more remote localities work less hours per week.^21^ However, we do not observe a similar gradient for HEWs – if anything, HEWs in more remote localities report working slightly *more* hours per month than their peers located closer to the woreda capital.^22^

Together, these results indicate that the total person-hours (number of extension workers and the hours they put in) in agricultural extension is lower in more remote localities compared to those located closer to the woreda capital. This alone could explain the finding in Section 5 according to which farm households in more remote kebeles are less likely to receive agriculture extension services.

**Fig. 3:**
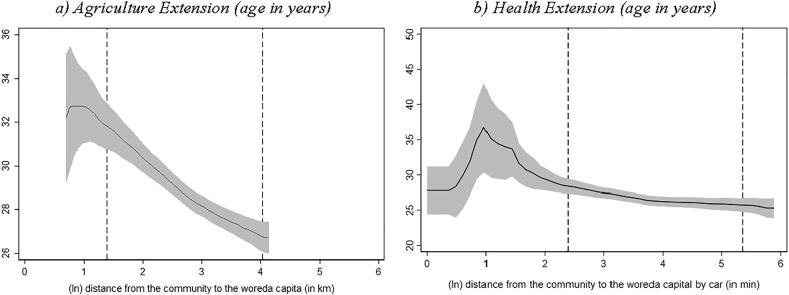
Age of the extension worker and distance to woreda capital Source: Authors’ calculation based on Digital Green’s DA survey, 2018 and Productive Safety Net Program (PSNP) survey, 2017. Note: Local polynomial regression. The vertical axis in both graphs measures the extension workers age in years. The area between the dashed lines indicates the 90% of the distance distribution (between the kebele center and woreda capital). (For interpretation of the references to colour in this figure legend, the reader is referred to the web version of this article.)

**Fig. 4:**
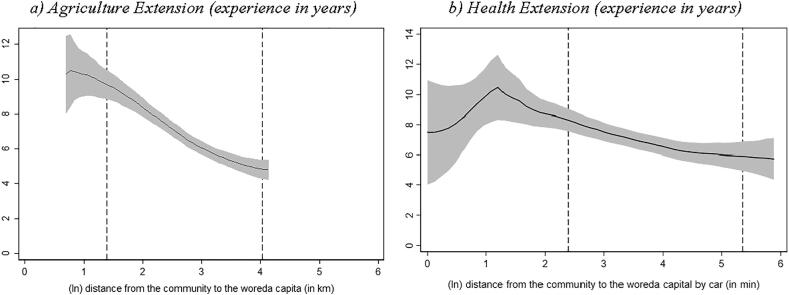
Work experience of extension worker and distance to woreda capital Source: Authors’ calculation based on Digital Green’s DA survey, 2018 and Productive Safety Net Program (PSNP) survey, 2017. Note: Local polynomial regression. The vertical axis in both graphs measures the extension worker's work experience in years. The area between the dashed lines indicates the 90% of the distance distribution (between the kebele center and woreda capital). (For interpretation of the references to colour in this figure legend, the reader is referred to the web version of this article.)

**Fig. 5:**
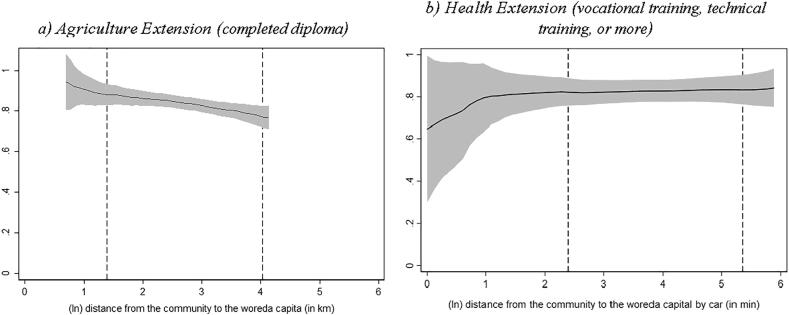
Education level of extension worker and distance to woreda capital Source: Authors’ calculation based on Digital Green’s DA survey, 2018 and Productive Safety Net Program (PSNP) survey, 2017. Note: Local polynomial regression. The vertical axis in graph a) measures the share of agriculture extension workers that reported to have completed a diploma or a higher level of education and graph b) the share of health extension workers that reported to have completed vocational/technical training or higher level of education. The area between the dashed lines indicates the 90% of the distance distribution (between the kebele center and woreda capital). (For interpretation of the references to colour in this figure legend, the reader is referred to the web version of this article.)

We used the same techniques to assess how some other key characteristics of the extension workers vary by remoteness. We see that both agriculture and health extension workers located in more remote localities are somewhat younger (Fig. 3) and less experienced (Fig. 4) than their peers in less remote localities. The likelihood that the agricultural extension worker had completed their diploma or achieved higher education declines as we move farther from the woreda capital (Fig. 5). However, such negative education gradient does not exist when we use our health extension survey data.^23^ These reported associations hold if we control for woreda fixed effects; see columns 3 to 5 in Tables A1 and A2 in the Appendix.

**Fig. 6:**
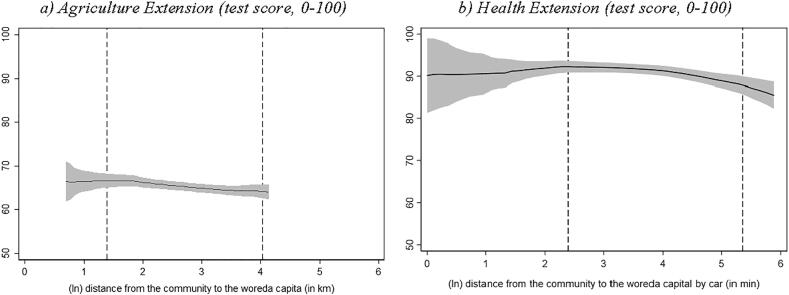
Knowledge level of extension worker and distance to woreda capital Source: Authors’ calculation based on Digital Green’s DA survey, 2018 and Productive Safety Net Program (PSNP) survey, 2017. Note: Local polynomial regression. The vertical axis in both graphs measures the job-related knowledge test score result (min = 0, max = 100) of the extension worker. The area between the dashed lines indicates the 90 percent interval of the distance distribution (between the kebele center and woreda capital). (For interpretation of the references to colour in this figure legend, the reader is referred to the web version of this article.)

Finally, in both surveys we assessed extension workers' work-related knowledge through quizzes (Fig. 6). For agricultural extension workers, these knowledge questions focused on growing practices of teff, maize and wheat, while for health extension workers, the questions were designed to measure their knowledge about age-appropriate infant and young child feeding practices. Fig. 6 shows that both types of extension workers located in more remote localities score fewer points in their knowledge test compared to their counterparts in less remote localities. The corresponding regression coefficient based on woreda fixed effects is reported in column 6 in Tables A1 and A2 in the Appendix.

Overall, the results in Fig. 3, Fig. 4, Fig. 5, Fig. 6 raise a concern that the *quality* of both agriculture and health extension services decline as we move from more connected areas to less connected areas, albeit more so for agricultural extension services.

## 7 Conclusions

Previous literature has quantified a sizable remoteness penalty along several welfare dimensions and largely attributed this finding to economic disadvantages and higher trade barriers. But another reason for these observed lower welfare outcomes could be limited service delivery in more remote localities. We explore this hypothesis by focusing on two types of remoteness: (1) distance from the household to service centers; and (2) distance from service centers and villages to district capital. Using detailed household level data from rural Ethiopia, we find that exposure to agriculture service delivery decreases as we move from more connected villages to less connected villages. But relative remoteness within village, i.e., the last mile, also matters as households farther away from village centers are less likely to have contact with extension agents. These disadvantages compound for *doubly* remote households (i.e., those located in remote kebeles and far away from the services centers within their villages) that are least likely to receive extension services.

In the health sector, the location of the village is not correlated with exposure to services. But here too, the last mile matters: households and pregnant mothers located farther away from the health posts are considerably less like to receive key health services than others. Interestingly, the results are relatively consistent across the different types of services provided by the extension agents. We take this to imply that the overall access to the extension agents is driving the documented associations between remoteness and access to extension services.

These differences between the two sectors could be due to the fact that more remote villages tend to have fewer agriculture extension workers who also put in fewer hours than their peers in more connected areas. We do not document similar associations in the health sector. We also provide suggestive evidence that the quality of extension services declines as we move farther away from the woreda center: both agriculture and health extension workers are, on average, younger, less experienced, educated, and knowledgeable in more remote kebeles compared to their peers working in less remote kebeles. While this finding is in line with earlier evidence in curative health care in lower income countries (Das and Gertler, 2007, Das et al., 2008, Leonard and Masatu, 2007), we are not aware of previous work documenting this for agricultural service provision.

This study has limitations. First, while both surveys are geographically widespread, they are not nationally representative, nor representative of the regions where they were administered. Second, while we control for a wide array of factors that may be correlated with both the exposure of a household to extension services and its remoteness, we cannot claim that we are documenting causal relations here. Third, due to the secondary nature of these data, the distance measures vary slightly across the two surveys. While the correlation between different distance measures is typically high, we cannot rule out the possibility that the some of the differences in our findings between the two sectors are due to this discrepancy in the survey instruments.

With these caveats in mind, our findings have important implications for policy and research. First, it has been shown that investments in road infrastructure have large impacts on economic activities and that rates of returns to, for example, rural road construction are often high (e.g., Jacoby, 2000, Stifel et al., 2016). However, these calculations typically ignore the benefits of better connectivity for improved public service delivery, including in the social sectors. More investments in infrastructure may lead to further improvements in such services and might therefore provide additional justification for infrastructure investments.

Finally, to ensure better inclusiveness of remote rural residents in their access to social services, a number of actions could be envisaged. A denser system of outreach could be aimed to cover especially the last mile. Extension agents from outside areas are often reluctant to settle in more remote places because living conditions are usually worse and because no additional benefits are provided for those working in these areas. Offering better financial incentives to public agents posted in more remote areas could be one policy option to reverse this. However, non-financial incentives may also be highly effective. For example, Ashraf et al. (2020) show how emphasizing career opportunities within the civil service sector during the recruitment process in Zambia led to a more qualified pool of rural health workers who also performed better on their job. Exploring these policy options in the rural Ethiopian context forms an important path for future research.

## Declaration of Competing Interest

The authors declare that they have no known competing financial interests or personal relationships that could have appeared to influence the work reported in this paper.

## Footnotes

1 We can think of one exception where remoteness is linked to positive economic outcomes. Nunn and Puga (2012) show how in Africa, rugged terrain provided protection against slave trade and its negative effects on long-term economic development.

2 Analysis of public extension services in Ethiopia has received considerable attention in the literature. Previous work has shown how poorest households are not well covered by the public services for health, education, and agriculture (Khan et al., 2014, Pankhurst and Tiumelissan, 2018). While some authors found evidence that there was a gender bias in agricultural service delivery in Ethiopia (Mogues et al., 2009, Ragasa et al., 2013), others did not (Khan et al., 2014, Buehren et al., 2019). Dercon et al. (2009) show that access to exten-sion led to consumption growth, but some find a lack of focus by agricultural extension agents to agricultural tasks (Berhanu and Poulton, 2004) and too much attention on modern input provision and less towards knowledge transfers (Berhane et al., 2018).

3 While Ethiopia's health extension program has not been rigorously evaluated, many consider the program a success (Wakabi, 2008, Bilal et al., 2011, Bradley et al., 2012, HEPCAPS1 Project Team, 2012, Workie and Ramana, 2013, Admasu et al., 2016, Yitayal et al., 2014, Wang et al., 2016).

4 Digital Green is a non-governmental organization that focuses on developing and testing technology-based solutions to help farmers in poor countries.

5 The original study did not have a household level pre-intervention, i.e., baseline, survey.

6 The woredas were purposefully selected to ensure the availability of a sufficient number of kebeles (≥9 kebeles) unexposed to video-mediated extension prior to a randomized control trial (RCT) experiment for the Digital Green evaluation.

7 In woredas with 9–15 kebeles, the study considered all the kebeles. In woredas with more than 15 kebeles, the study randomly chose 15 kebeles. The reason for selecting such a large number of kebeles from each woreda was based on statistical power calculations, combined with the fact that we could not increase the number of woredas.

8 Development groups are a semi-formal administrative structure below kebele that comprise 25–30 farm households and serve as a grassroots forum to discuss local development issues.

9 We listed all the development groups by distance to the FTC and randomly chose five from those below the median distance and another five from above the median distance. For the survey, we again listed the 10 development groups by distance and randomly chose 1 from the closest, 1 from average distance, and another 1 from the farthest distance.

10 A follow-up survey with the same households was administered in August 2017, but these data are not used here.

11 PSNP is a geographically widespread safety net program that provides cash and in-kind transfers to approximately eight million people residing in chronically food insecure areas of rural Ethiopia. The two main components are a public works program for households with labor capacity and a direct support element that provides direct transfers to households without sufficient labor capacity.

12 This latter group served as a control group in the evaluation project. During listing, we asked all households to place themselves on to a poverty ladder that had 7 rungs. The first rung represented the very poorest households in the village and the highest (7th) rung the very richest households in the village. Households for the comparison (or control) sample were chosen from the bottom four rungs of the ranking.

13 The reasons for not interviewing all 264 HEWs were because the kebele did not have a health post or because the health extension worker was temporarily absent due to training, leave, or some other reason.

14 We report the estimates based on different sub-sets of these control variables in online Appendix C. Results are remarkably robust.

15 Relative distances are likely to be more important than absolute distances in this context. As the managers at the woreda agriculture or health bureaus are (directly or indirectly) responsible for assigning DAs and HEWs to the specific kebeles and make assignment decisions on the pool at their disposal in the woreda, relative distances from the center of the woreda – as reflected by terciles – seem the most sensible measure of remoteness. Similarly, within kebeles, HEWs and DAs may allocate more attention to households that are relatively closer to them in terms of distance.

16 We also considered Conley (1999) standard errors that robust to both spatial autocorrelation and heteroskedasticity. The estimated Conley standard errors are similar to the clustered standard errors reported here; see online Appendix D.

17 The estimate is statistically significant only at the 10 percent level, the p-value exceeds the Bonferroni-adjusted critical value based on a 0.10 significance level.

18 While the estimate is statistically significant at the 5 percent level and the p-value exceeds the Bonferroni-adjusted critical value based on 0.10 significance level.

19 The joint estimate is computed by summing up the coefficients on two 'Farthest tercile' coefficients in column 2; −0.064+(−0.074) = −0.138. Based on an F-test, this joint-estimate is statistically different from zero (p<0.01).

20 Column 1 in Tables E1 and E2 in the online appendix confirm the patterns reported in Fig. 1.

21 We asked the DAs to report the number of days they work in a typical week and also the number of hours they work in a typical day. Using these responses, we constructed a variable measuring the number of hours the DAs work per week. The HEW questionnaire contained similar questions, but the recall period was a month, i.e., "How many days in a month do you usually work as HEW?".

22 Column 2 in Table E1 in the online appendix confirms the negative slope observed in Fig. 2a. However, the estimated coefficients for health extension workers are statistically not different from zero, indicating that the positive slope observed in Fig. 2b is not robust to controlling for observed and unobserved woreda characteristics.

23 Note that the education measures are not fully comparable across the two surveys. For agriculture extension workers, we use a variable that assumes a value of 1 (and zero otherwise) if the DA reported to have completed a diploma or a higher level of education, whereas for the HEWs the binary variable is determined based on them completing vocational training, technical training, or a higher level of education.
