# Supplementary material for: Geography of public service delivery in rural Ethiopia
Source: World Dev. 2020 Dec;136:105133. doi: 10.1016/j.worlddev.2020.105133 (PMC7511693; doi:10.1016/j.worlddev.2020.105133)
Supplement: Supplementary data 1 [file mmc1.docx]

# **Online appendix material for:**

# **Geography of public service delivery in rural Ethiopia**

**Contents:**

[Appendix A. Survey districts (woredas) 2](#_Toc38287162)

[Appendix B. Distributions of the distance measures 3](#_Toc38287163)

[Appendix C. Different specifications, Table 5 & 6 4](#_Toc38287164)

[Appendix D. Conley standard errors 9](#_Toc38287165)

[Appendix E. DA and HEW characteristics by remoteness 15](#_Toc38287166)

## Appendix A. Survey districts (woredas)

The two maps below show the districts (woredas) included in the Digital green survey (Figure A1) and PSNP evaluation survey (Figure A2).

Figure A1: Woredas included in the Digital Green evaluation survey


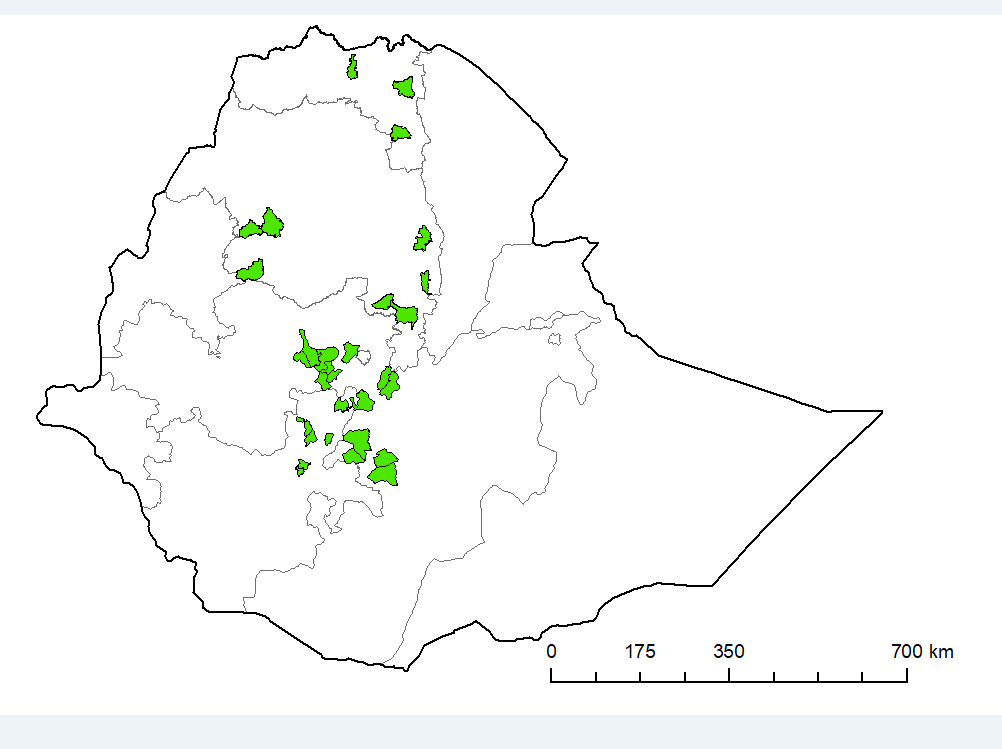


Figure A2: Woredas included in the PSNP evaluation survey


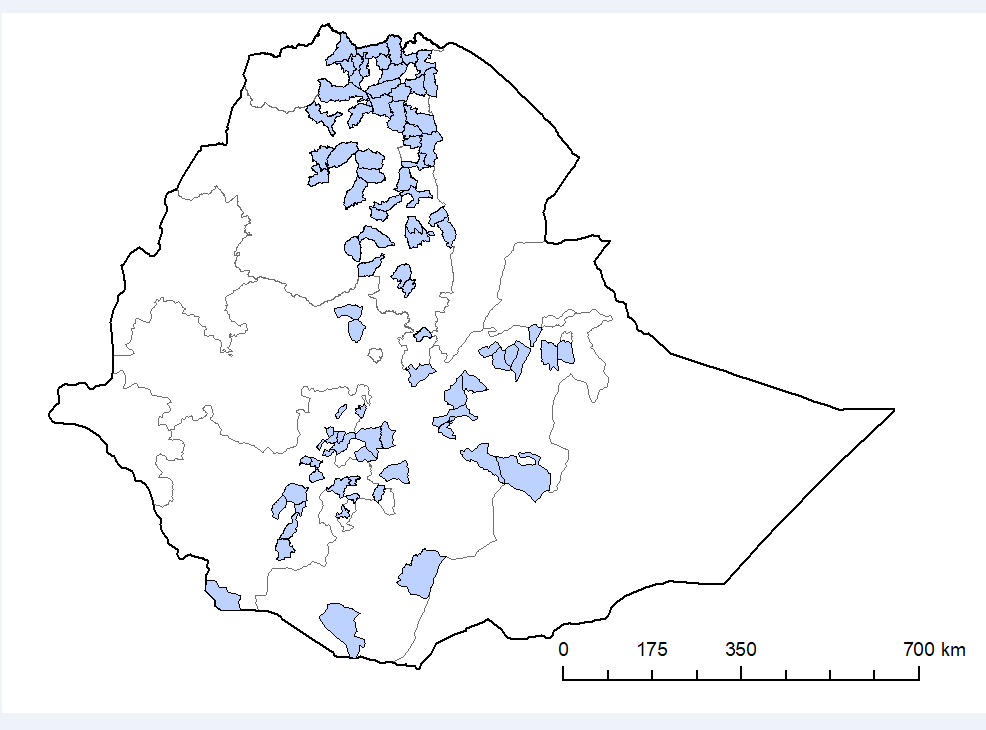


## Appendix B. Distributions of the distance measures

Figure B1: Histogram of the distance measures – agriculture extension

| *a) Distance from kebele to woreda capital (in km)* | *b) Travel time from household to FTC (in minutes)* |
| --- | --- |
|  | 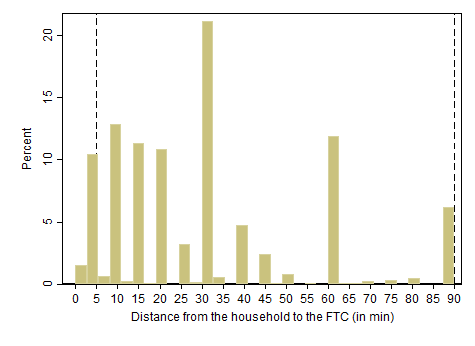 |

Source: Authors’ calculation based on Digital Green’s household and DA survey, 2017 and 2018.

Note: The area between the dashed lines indicates the 90% of the distance distribution

Figure B2: Histogram of the distance measures – health extension

| *a) Travel time from kebele to woreda capital (in minutes)* | *b) Travel time from household to health post (in minutes)* |
| --- | --- |
| 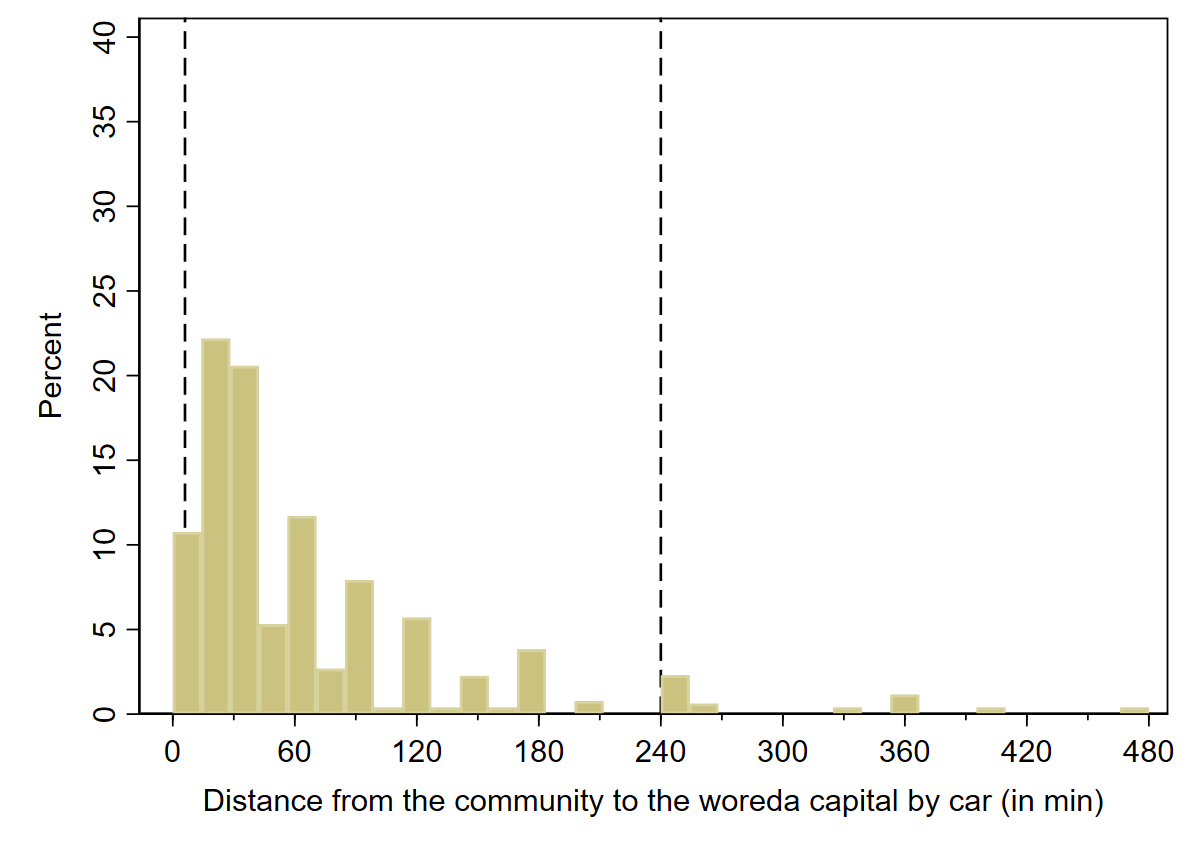 | 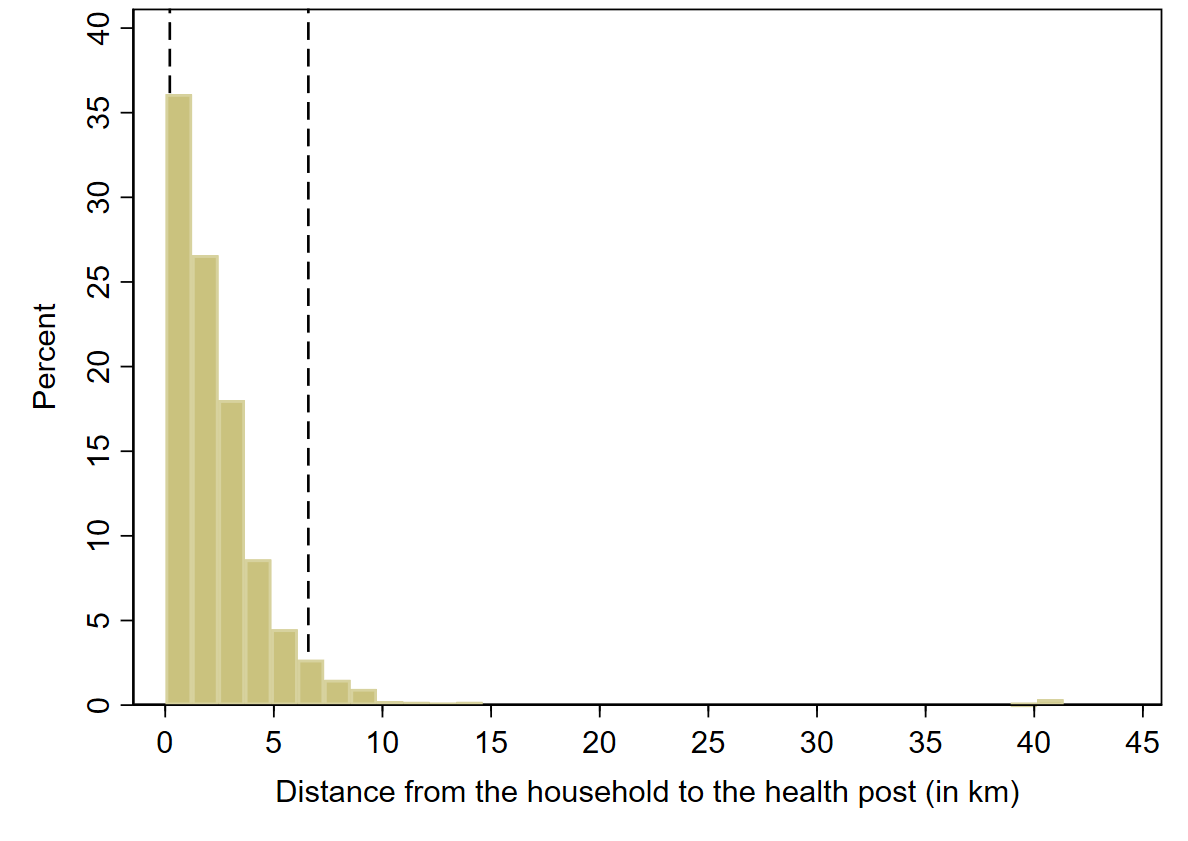 |

Source: Authors’ calculation based on the 2017 Productive Safety Net Program (PSNP) survey data.

Note: The area between the dashed lines indicates the 90% of the distance distribution.

## Appendix C. Different specifications, Table 5 & 6

Tables C1 and C2 below report the estimates based on different sub-sets of control variables. In the first of the three columns, we only control for region fixed effects (binary capturing each region). In the second, we add household level controls listed in Section 4 in the main text. The third column reproduces the estimates reported in Tables 5 and 6 based on Equation (1). The coefficients are qualitatively similar across all three specifications.

Table C1: Different specifications of Table 5

| *Outcome variable:* | *Was this plot visited by the Develop-ment Agent (DA)?* | | | *Did the DA advice on amount of fertilizer to use?* | | | *Did the DA advice on type of fertilizer to use?* | | | |
| --- | --- | --- | --- | --- | --- | --- | --- | --- | --- | --- |
| Distance to woreda from kebele center (km): | | | | | | | | | | |
| First tercile | (reference) | (reference) | (reference) | (reference) | (reference) | (reference) | (reference) | (reference) | (reference) |  |
|  |  |  |  |  |  |  |  |  |  |  |
| Second tercile | -0.037 | -0.028 | -0.024 | -0.063* | -0.047 | -0.047 | -0.066* | -0.054 | -0.051 |  |
|  | (0.032) | (0.032) | (0.032) | (0.035) | (0.037) | (0.035) | (0.035) | (0.036) | (0.034) |  |
| Furthest tercile | -0.066** | -0.051* | -0.051* | -0.082** | -0.061* | -0.064* | -0.095*** | -0.076** | -0.080** |  |
|  | (0.031) | (0.030) | (0.029) | (0.032) | (0.033) | (0.032) | (0.033) | (0.033) | (0.031) |  |
| Household's travel time to FTC (in minutes): | | | | | | | | | | |
| First tercile | (reference) | (reference) | (reference) | (reference) | (reference) | (reference) | (reference) | (reference) | (reference) |  |
|  |  |  |  |  |  |  |  |  |  |  |
| Second FTC tercile | -0.092*** | -0.079*** | -0.084*** | -0.068** | -0.056* | -0.054** | -0.065** | -0.052* | -0.049* |  |
|  | (0.026) | (0.025) | (0.026) | (0.029) | (0.028) | (0.026) | (0.029) | (0.028) | (0.026) |  |
| Furthest FTC tercile | -0.128*** | -0.109*** | -0.109*** | -0.094*** | -0.076*** | -0.074*** | -0.087*** | -0.069** | -0.067** |  |
|  | (0.027) | (0.027) | (0.028) | (0.025) | (0.025) | (0.025) | (0.026) | (0.027) | (0.027) |  |
| Region dummies? | Yes | Yes | Yes | Yes | Yes | Yes | Yes | Yes | Yes |  |
| Household level controls? | No | Yes | Yes | No | Yes | Yes | No | Yes | Yes |  |
| Woreda fixed effects? | No | No | Yes | No | No | Yes | No | No | Yes |  |
| Number of observations | 2,367 | 2,367 | 2,367 | 2,367 | 2,367 | 2,367 | 2,367 | 2,367 | 2,367 |  |
| R^2^ | 0.192 | 0.226 | 0.065 | 0.184 | 0.216 | 0.051 | 0.181 | 0.215 | 0.051 |  |
| Adjusted R^2^ | 0.190 | 0.220 | 0.058 | 0.181 | 0.209 | 0.043 | 0.179 | 0.207 | 0.043 |  |

**Table C1: Different specifications of Table 5 – Continued**

| *Outcome variable:* | *Did the DA advice on type of seed to use on this plot?* | | | *Did a DA advice on the type of crop suitable to this plot?* | | | *Did the DA provide crop specific advice on the plot?* | | |
| --- | --- | --- | --- | --- | --- | --- | --- | --- | --- |
| Distance to woreda from kebele center (km): | | | | | | | | | |
| First tercile | (reference) | (reference) | (reference) | (reference) | (reference) | (reference) | (reference) | (reference) | (reference) |
|  |  |  |  |  |  |  |  |  |  |
| Second tercile | -0.079** | -0.066* | -0.066* | -0.047 | -0.034 | -0.033 | -0.005 | 0.004 | -0.000 |
|  | (0.037) | (0.037) | (0.035) | (0.031) | (0.032) | (0.030) | (0.033) | (0.033) | (0.032) |
| Furthest tercile | -0.112*** | -0.092*** | -0.096*** | -0.077** | -0.061* | -0.065** | -0.045 | -0.034 | -0.032 |
|  | (0.032) | (0.031) | (0.030) | (0.033) | (0.032) | (0.031) | (0.029) | (0.030) | (0.028) |
| Household's travel time to FTC (in minutes): | | | | | | | | | |
| First tercile | (reference) | (reference) | (reference) | (reference) | (reference) | (reference) | (reference) | (reference) | (reference) |
|  |  |  |  |  |  |  |  |  |  |
| Second FTC tercile | -0.079** | -0.065** | -0.067** | -0.081** | -0.069** | -0.070** | -0.051* | -0.043* | -0.039 |
|  | (0.030) | (0.028) | (0.025) | (0.035) | (0.034) | (0.034) | (0.026) | (0.024) | (0.023) |
| Furthest FTC tercile | -0.097*** | -0.076*** | -0.075*** | -0.090*** | -0.076*** | -0.075*** | -0.065** | -0.054** | -0.048* |
|  | (0.026) | (0.026) | (0.026) | (0.024) | (0.026) | (0.026) | (0.024) | (0.025) | (0.024) |
| Region dummies? | Yes | Yes | Yes | Yes | Yes | Yes | Yes | Yes | Yes |
| Household level controls? | No | Yes | Yes | No | Yes | Yes | No | Yes | Yes |
| Woreda fixed effects? | No | No | Yes | No | No | Yes | No | No | Yes |
| Number of observations | 2,367 | 2,367 | 2,367 | 2,367 | 2,367 | 2,367 | 2,369 | 2,368 | 2,368 |
| R^2^ | 0.177 | 0.212 | 0.062 | 0.159 | 0.185 | 0.046 | 0.225 | 0.248 | 0.033 |
| Adjusted R^2^ | 0.175 | 0.204 | 0.054 | 0.157 | 0.177 | 0.039 | 0.222 | 0.241 | 0.025 |

Source: Authors’ calculation based on Digital Green’s household and DA survey, 2017 and 2018.

Notes: See Table 5 in the main text.

Table C2: Different specifications of Table 6

| *Outcome variable:* | *Knows a HEW working in the kebele* | | | *Has met with HEW in the last 3 months* | | | *HEW ever visited home* | | |
| --- | --- | --- | --- | --- | --- | --- | --- | --- | --- |
| Distance to woreda from kebele center (km): | | | | | | | | | |
| First tercile | (reference) | (reference) | (reference) | (reference) | (reference) | (reference) | (reference) | (reference) | (reference) |
|  |  |  |  |  |  |  |  |  |  |
| Second tercile | -0.011 | -0.013 | -0.005 | 0.030 | 0.028 | 0.027 | 0.018 | 0.015 | -0.001 |
|  | (0.025) | (0.025) | (0.027) | (0.024) | (0.024) | (0.023) | (0.024) | (0.024) | (0.023) |
| Farthest tercile | -0.001 | 0.002 | -0.019 | 0.004 | 0.008 | -0.004 | -0.001 | -0.000 | -0.013 |
|  | (0.029) | (0.028) | (0.024) | (0.026) | (0.027) | (0.027) | (0.026) | (0.026) | (0.026) |
| Household's distance to health post (km): | | | | | | | | | |
| First tercile | (reference) | (reference) | (reference) | (reference) | (reference) | (reference) | (reference) | (reference) | (reference) |
|  |  |  |  |  |  |  |  |  |  |
| Second tercile | -0.018 | -0.018 | -0.018 | -0.050** | -0.046** | -0.048** | -0.026 | -0.024 | -0.025 |
|  | (0.019) | (0.018) | (0.018) | (0.022) | (0.022) | (0.022) | (0.020) | (0.020) | (0.020) |
| Farthest tercile | -0.065*** | -0.065*** | -0.066*** | -0.080*** | -0.076*** | -0.078*** | -0.044** | -0.044** | -0.045** |
|  | (0.020) | (0.020) | (0.019) | (0.022) | (0.022) | (0.022) | (0.020) | (0.020) | (0.020) |
| Region dummies? | Yes | Yes | Yes | Yes | Yes | Yes | Yes | Yes | Yes |
| Household level controls? | No | Yes | Yes | No | Yes | Yes | No | Yes | Yes |
| Woreda fixed effects? | No | No | Yes | No | No | Yes | No | No | Yes |
| Observations | 2615 | 2615 | 2615 | 2615 | 2615 | 2615 | 2615 | 2615 | 2615 |
| *R*^2^ | 0.076 | 0.096 | 0.022 | 0.058 | 0.071 | 0.018 | 0.053 | 0.066 | 0.016 |
| Adjusted *R*^2^ | 0.074 | 0.087 | 0.013 | 0.056 | 0.062 | 0.009 | 0.051 | 0.057 | 0.008 |

**Table C2: Different specifications of Table 6 – Continued**

| *Outcome variable:* | *HEW visited during pregnancy* | | | *Received ante-natal care during pregnancy* | | |
| --- | --- | --- | --- | --- | --- | --- |
| Distance to woreda from kebele center (km): | | | | | | |
| First tercile | (reference) | (reference) | (reference) | (reference) | (reference) | (reference) |
|  |  |  |  |  |  |  |
| Second tercile | -0.008 | -0.009 | -0.025 | -0.017 | -0.017 | -0.012 |
|  | (0.024) | (0.025) | (0.025) | (0.025) | (0.024) | (0.024) |
|  |  |  |  |  |  |  |
| Farthest tercile | -0.007 | -0.005 | -0.019 | 0.009 | 0.008 | -0.002 |
|  | (0.030) | (0.030) | (0.030) | (0.026) | (0.025) | (0.024) |
| Household's distance to health post (km): | | | | | | |
| First tercile | (reference) | (reference) | (reference) | (reference) | (reference) | (reference) |
|  |  |  |  |  |  |  |
| Second tercile | -0.029 | -0.028 | -0.030 | -0.012 | -0.012 | -0.012 |
|  | (0.019) | (0.019) | (0.019) | (0.019) | (0.019) | (0.019) |
| Farthest tercile | -0.048*** | -0.047** | -0.048** | -0.043** | -0.043** | -0.043** |
|  | (0.018) | (0.018) | (0.019) | (0.019) | (0.020) | (0.019) |
| Region dummies? | Yes | Yes | Yes | Yes | Yes | Yes |
| Household level controls? | No | Yes | Yes | No | Yes | Yes |
| Woreda fixed effects? | No | No | Yes | No | No | Yes |
| Observations | 2615 | 2615 | 2615 | 2615 | 2615 | 2615 |
| *R*^2^ | 0.068 | 0.076 | 0.013 | 0.069 | 0.085 | 0.011 |
| Adjusted *R*^2^ | 0.065 | 0.067 | 0.005 | 0.066 | 0.076 | 0.002 |

Source: Authors’ calculation based on the 2017 Productive Safety Net Program (PSNP) survey data.

Notes: See Table 6 in the main text.

## Appendix D. Conley standard errors

Tables D1 and D2 replicate Tables 5 and 6 in the main text, respectively, but based on Conley (1999) standard errors. Conley's approach accounts for spatial correlation across woredas until a certain distance threshold. Using the woreda centroid coordinates we calculated that the mean distance between the Digital Green woredas is 280 km (min: 14 km & max 803 km) and between the PSNP woredas 417 km (min 1.3 km and max: 1,170 km). Considering these distances and assuming that correlation between woredas that are farther than 100 km apart is unlikely, we set the distance cut-off points at 25 km, 50 km, and 100 km. We see that the standard errors are very similar to the original, clustered ones reported in Tables 5 and 6.

**Table D1: Conley standard errors for Table 5 in the main text**

**Column 1: Was this plot visited by the Development Agent (DA)?**

| ***distance cut-off:*** | ***5 km*** | ***25 km*** | ***50 km*** | ***100 km*** | ***Table 5*** |
| --- | --- | --- | --- | --- | --- |
| Distance to woreda from kebele center (km): | | | | | |
| First tercile | (reference) | (reference) | (reference) | (reference) | (reference) |
|  |  |  |  |  |  |
| Second tercile | -0.024 | -0.024 | -0.024 | -0.024 | -0.024 |
|  | (0.031) | (0.031) | (0.031) | (0.028) | (0.032) |
| Farthest tercile | -0.051* | -0.051* | -0.051* | -0.051* | -0.051* |
|  | (0.029) | (0.030) | (0.030) | (0.027) | (0.029) |
| Household's travel time to FTC (in minutes): | | | | | |
| First tercile | (reference) | (reference) | (reference) | (reference) | (reference) |
|  |  |  |  |  |  |
| Second tercile | -0.084*** | -0.084*** | -0.084*** | -0.084*** | -0.084*** |
|  | (0.026) | (0.026) | (0.027) | (0.029) | (0.026) |
| Farthest tercile | -0.109*** | -0.109*** | -0.109*** | -0.109*** | -0.109*** |
|  | (0.028) | (0.027) | (0.028) | (0.030) | (0.028) |

Source: Authors’ calculation based on Digital Green’s household and DA survey, 2017 and 2018.

**Column 2: Did the DA give advice on amount of fertilizer to use?**

| ***distance cut-off:*** | ***5 km*** | ***25 km*** | ***50 km*** | ***100 km*** | ***Table 5*** |
| --- | --- | --- | --- | --- | --- |
| Distance to woreda from kebele center (km): | | | | | |
| First tercile | (reference) | (reference) | (reference) | (reference) | (reference) |
|  |  |  |  |  |  |
| Second tercile | -0.047 | -0.047 | -0.047 | -0.047 | -0.047 |
|  | (0.034) | (0.034) | (0.035) | (0.036) | (0.035) |
| Farthest tercile | -0.064** | -0.064** | -0.064** | -0.064*** | -0.064* |
|  | (0.031) | (0.030) | (0.028) | (0.025) | (0.032) |
| Household's travel time to FTC (in minutes): | | | | | |
| First tercile | (reference) | (reference) | (reference) | (reference) | (reference) |
|  |  |  |  |  |  |
| Second tercile | -0.054** | -0.054** | -0.054** | -0.054* | -0.054** |
|  | (0.026) | (0.027) | (0.028) | (0.030) | (0.026) |
| Farthest tercile | -0.074*** | -0.074*** | -0.074*** | -0.074*** | -0.074*** |
|  | (0.024) | (0.025) | (0.024) | (0.022) | (0.025) |

Source: Authors’ calculation based on Digital Green’s household and DA survey, 2017 and 2018.

**Column 3: Did the DA give advice on type of fertilizer to use?**

| ***distance cut-off:*** | ***5 km*** | ***25 km*** | ***50 km*** | ***100 km*** | ***Table 5*** |
| --- | --- | --- | --- | --- | --- |
| Distance to woreda from kebele center (km): | | | | | |
| First tercile | (reference) | (reference) | (reference) | (reference) | (reference) |
|  |  |  |  |  |  |
| Second tercile | -0.051 | -0.051 | -0.051 | -0.051 | -0.051 |
|  | (0.034) | (0.034) | (0.035) | (0.037) | (0.034) |
| Farthest tercile | -0.080*** | -0.080*** | -0.080*** | -0.080*** | -0.080** |
|  | (0.031) | (0.030) | (0.030) | (0.028) | (0.031) |
| Household's travel time to FTC (in minutes): | | | | | |
| First tercile | (reference) | (reference) | (reference) | (reference) | (reference) |
|  |  |  |  |  |  |
| Second tercile | -0.049* | -0.049* | -0.049* | -0.049 | -0.049* |
|  | (0.026) | (0.027) | (0.029) | (0.032) | (0.026) |
| Farthest tercile | -0.067** | -0.067** | -0.067** | -0.067*** | -0.067** |
|  | (0.027) | (0.027) | (0.026) | (0.024) | (0.027) |

Source: Authors’ calculation based on Digital Green’s household and DA survey, 2017 and 2018.

**Column 4: Did the DA give advice on type of seed to use on this plot?**

| ***distance cut-off:*** | ***5 km*** | ***25 km*** | ***50 km*** | ***100 km*** | ***Table 5*** |
| --- | --- | --- | --- | --- | --- |
| Distance to woreda from kebele center (km): | | | | | |
| First tercile | (reference) | (reference) | (reference) | (reference) | (reference) |
|  |  |  |  |  |  |
| Second tercile | -0.066* | -0.066* | -0.066* | -0.066* | -0.066* |
|  | (0.034) | (0.035) | (0.036) | (0.037) | (0.035) |
| Farthest tercile | -0.096*** | -0.096*** | -0.096*** | -0.096*** | -0.096*** |
|  | (0.030) | (0.030) | (0.028) | (0.025) | (0.030) |
| Household's travel time to FTC (in minutes): | | | | | |
| First tercile | (reference) | (reference) | (reference) | (reference) | (reference) |
|  |  |  |  |  |  |
| Second tercile | -0.067*** | -0.067*** | -0.067** | -0.067** | -0.067** |
|  | (0.025) | (0.026) | (0.027) | (0.030) | (0.025) |
| Farthest tercile | -0.075*** | -0.075*** | -0.075*** | -0.075*** | -0.075*** |
|  | (0.026) | (0.026) | (0.026) | (0.026) | (0.026) |

Source: Authors’ calculation based on Digital Green’s household and DA survey, 2017 and 2018.

**Column 5: Did a DA give advice on the type of crop suitable to this plot?**

| ***distance cut-off:*** | ***5 km*** | ***25 km*** | ***50 km*** | ***100 km*** | ***Table 5*** |
| --- | --- | --- | --- | --- | --- |
| Distance to woreda from kebele center (km): | | | | | |
| First tercile | (reference) | (reference) | (reference) | (reference) | (reference) |
|  |  |  |  |  |  |
| Second tercile | -0.033 | -0.033 | -0.033 | -0.033 | -0.033 |
|  | (0.030) | (0.030) | (0.030) | (0.029) | (0.030) |
| Farthest tercile | -0.065** | -0.065** | -0.065** | -0.065*** | -0.065** |
|  | (0.030) | (0.029) | (0.028) | (0.025) | (0.031) |
| Household's travel time to FTC (in minutes): | | | | | |
| First tercile | (reference) | (reference) | (reference) | (reference) | (reference) |
|  |  |  |  |  |  |
| Second tercile | -0.070** | -0.070** | -0.070** | -0.070* | -0.070** |
|  | (0.033) | (0.034) | (0.035) | (0.037) | (0.034) |
| Farthest tercile | -0.075*** | -0.075*** | -0.075*** | -0.075*** | -0.075*** |
|  | (0.026) | (0.026) | (0.024) | (0.021) | (0.026) |

Source: Authors’ calculation based on Digital Green’s household and DA survey, 2017 and 2018.

**Column 6: Did the DA provide crop specific advice on the plot?**

| ***distance cut-off:*** | ***5 km*** | ***25 km*** | ***50 km*** | ***100 km*** | ***Table 5*** |
| --- | --- | --- | --- | --- | --- |
| Distance to woreda from kebele center (km): | | | | | |
| First tercile | (reference) | (reference) | (reference) | (reference) | (reference) |
|  |  |  |  |  |  |
| Second tercile | -0.000 | -0.000 | -0.000 | -0.000 | -0.000 |
|  | (0.032) | (0.031) | (0.032) | (0.032) | (0.032) |
| Farthest tercile | -0.032 | -0.032 | -0.032 | -0.032 | -0.032 |
|  | (0.028) | (0.028) | (0.028) | (0.032) | (0.028) |
| Household's travel time to FTC (in minutes): | | | | | |
| First tercile | (reference) | (reference) | (reference) | (reference) | (reference) |
|  |  |  |  |  |  |
| Second tercile | -0.039* | -0.039* | -0.039* | -0.039 | -0.039 |
|  | (0.023) | (0.022) | (0.023) | (0.024) | (0.023) |
| Farthest tercile | -0.048** | -0.048** | -0.048** | -0.048** | -0.048* |
|  | (0.024) | (0.024) | (0.022) | (0.021) | (0.024) |

Source: Authors’ calculation based on Digital Green’s household and DA survey, 2017 and 2018.

Table D2: Conley standard errors for Table 6 in the main text

**Column 1: Knows a HEW working in the kebele**

| ***distance cut-off:*** | ***5 km*** | ***25 km*** | ***50 km*** | ***100 km*** | ***Table 6*** |
| --- | --- | --- | --- | --- | --- |
| Distance to woreda from kebele center (km): | | | | | |
| First tercile | (reference) | (reference) | (reference) | (reference) | (reference) |
|  |  |  |  |  |  |
| Second tercile | -0.005 | -0.005 | -0.005 | -0.005 | -0.005 |
|  | (0.027) | (0.027) | (0.028) | (0.028) | (0.027) |
| Farthest tercile | -0.019 | -0.019 | -0.019 | -0.019 | -0.019 |
|  | (0.024) | (0.024) | (0.025) | (0.022) | (0.024) |
| Household's distance to health post (km): | | | | | |
| First tercile | (reference) | (reference) | (reference) | (reference) | (reference) |
|  |  |  |  |  |  |
| Second tercile | -0.018 | -0.018 | -0.018 | -0.018 | -0.018 |
|  | (0.018) | (0.018) | (0.019) | (0.018) | (0.018) |
| Farthest tercile | -0.066*** | -0.066*** | -0.066*** | -0.066*** | -0.066*** |
|  | (0.019) | (0.019) | (0.019) | (0.020) | (0.019) |

Source: Authors’ calculation based on the 2017 Productive Safety Net Program (PSNP) survey data.

**Column 2: Has met with HEW in the last 3 months?**

| ***distance cut-off:*** | ***5 km*** | ***25 km*** | ***50 km*** | ***100 km*** | ***Table 6*** |
| --- | --- | --- | --- | --- | --- |
| Distance to woreda from kebele center (km): | | | | | |
| First tercile | (reference) | (reference) | (reference) | (reference) | (reference) |
|  |  |  |  |  |  |
| Second tercile | 0.027 | 0.027 | 0.027 | 0.027 | 0.027 |
|  | (0.022) | (0.023) | (0.025) | (0.027) | (0.023) |
| Farthest tercile | -0.004 | -0.004 | -0.004 | -0.004 | -0.004 |
|  | (0.027) | (0.026) | (0.025) | (0.027) | (0.027) |
| Household's distance to health post (km): | | | | | |
| First tercile | (reference) | (reference) | (reference) | (reference) | (reference) |
|  |  |  |  |  |  |
| Second tercile | -0.048** | -0.048** | -0.048** | -0.048** | -0.048** |
|  | (0.022) | (0.022) | (0.022) | (0.023) | (0.022) |
| Farthest tercile | -0.078*** | -0.078*** | -0.078*** | -0.078*** | -0.078*** |
|  | (0.022) | (0.021) | (0.021) | (0.022) | (0.022) |

Source: Authors’ calculation based on the 2017 Productive Safety Net Program (PSNP) survey data.

**Column 3: HEW ever visited home?**

| ***distance cut-off:*** | ***5 km*** | ***25 km*** | ***50 km*** | ***100 km*** | ***Table 6*** |
| --- | --- | --- | --- | --- | --- |
| Distance to woreda from kebele center (km): | | | | | |
| First tercile | (reference) | (reference) | (reference) | (reference) | (reference) |
|  |  |  |  |  |  |
| Second tercile | -0.001 | -0.001 | -0.001 | -0.001 | -0.001 |
|  | (0.023) | (0.023) | (0.022) | (0.020) | (0.023) |
| Farthest tercile | -0.013 | -0.013 | -0.013 | -0.013 | -0.013 |
|  | (0.026) | (0.026) | (0.025) | (0.021) | (0.026) |
| Household's distance to health post (km): | | | | | |
| First tercile | (reference) | (reference) | (reference) | (reference) | (reference) |
|  |  |  |  |  |  |
| Second tercile | -0.025 | -0.025 | -0.025 | -0.025 | -0.025 |
|  | (0.020) | (0.019) | (0.019) | (0.019) | (0.020) |
| Farthest tercile | -0.045** | -0.045** | -0.045** | -0.045** | -0.045** |
|  | (0.019) | (0.019) | (0.019) | (0.020) | (0.020) |

Source: Authors’ calculation based on the 2017 Productive Safety Net Program (PSNP) survey data.

**Column 4: HEW visited during pregnancy?**

| ***distance cut-off:*** | ***5 km*** | ***25 km*** | ***50 km*** | ***100 km*** | ***Table 6*** |
| --- | --- | --- | --- | --- | --- |
| Distance to woreda from kebele center (km): | | | | | |
| First tercile | (reference) | (reference) | (reference) | (reference) | (reference) |
|  |  |  |  |  |  |
| Second tercile | -0.025 | -0.025 | -0.025 | -0.025 | -0.025 |
|  | (0.024) | (0.024) | (0.023) | (0.022) | (0.025) |
| Farthest tercile | -0.019 | -0.019 | -0.019 | -0.019 | -0.019 |
|  | (0.030) | (0.030) | (0.028) | (0.025) | (0.030) |
| Household's distance to health post (km): | | | | | |
| First tercile | (reference) | (reference) | (reference) | (reference) | (reference) |
|  |  |  |  |  |  |
| Second tercile | -0.030 | -0.030 | -0.030* | -0.030* | -0.030 |
|  | (0.019) | (0.018) | (0.017) | (0.016) | (0.019) |
| Farthest tercile | -0.048*** | -0.048*** | -0.048*** | -0.048*** | -0.048** |
|  | (0.018) | (0.018) | (0.017) | (0.016) | (0.019) |

Source: Authors’ calculation based on the 2017 Productive Safety Net Program (PSNP) survey data.

**Column 5: Received antenatal care?**

| ***distance cut-off:*** | ***5 km*** | ***25 km*** | ***50 km*** | ***100 km*** | ***Table 6*** |
| --- | --- | --- | --- | --- | --- |
| Distance to woreda from kebele center (km): | | | | | |
| First tercile | (reference) | (reference) | (reference) | (reference) | (reference) |
|  |  |  |  |  |  |
| Second tercile | -0.012 | -0.012 | -0.012 | -0.012 | -0.012 |
|  | (0.024) | (0.024) | (0.023) | (0.025) | (0.024) |
| Farthest tercile | -0.002 | -0.002 | -0.002 | -0.002 | -0.002 |
|  | (0.024) | (0.024) | (0.023) | (0.023) | (0.024) |
| Household's distance to health post (km): | | | | | |
| First tercile | (reference) | (reference) | (reference) | (reference) | (reference) |
|  |  |  |  |  |  |
| Second tercile | -0.012 | -0.012 | -0.012 | -0.012 | -0.012 |
|  | (0.018) | (0.018) | (0.019) | (0.019) | (0.019) |
| Farthest tercile | -0.043** | -0.043** | -0.043** | -0.043** | -0.043** |
|  | (0.019) | (0.019) | (0.019) | (0.018) | (0.019) |

Source: Authors’ calculation based on the 2017 Productive Safety Net Program (PSNP) survey data.

## Appendix E. DA and HEW characteristics by remoteness

Table E1: Development Agent (DA) characteristics by remoteness

|  | (1) | (2) | (3) | (4) | (5) | (6) |
| --- | --- | --- | --- | --- | --- | --- |
| *Outcome variable:* | *number of DAs in the kebele* | *working hours per week* | *age in years* | *work experience (years)* | *education* | *test score* |
| Distance to woreda from kebele center (in minutes) | | | | | | |
| First tercile | (reference) | (reference) | (reference) | (reference) | (reference) | (reference) |
|  |  |  |  |  |  |  |
| Second tercile | -0.026 | -3.149** | -2.255*** | -2.202*** | -0.032 | -1.837** |
|  | (0.125) | (1.353) | (0.589) | (0.539) | (0.028) | (0.815) |
| Farthest tercile | -0.459*** | -3.104*** | -3.099*** | -2.959*** | -0.068* | -2.796*** |
|  | (0.100) | (1.053) | (0.610) | (0.523) | (0.035) | (0.908) |
| Woreda fixed effects? | Yes | Yes | Yes | Yes | Yes | Yes |
| Observations | 781 | 781 | 781 | 781 | 781 | 781 |
| Within-R^2^ | 0.045 | 0.018 | 0.058 | 0.080 | 0.007 | 0.014 |

Source: Authors’ calculation based on Digital Green’s household and DA survey, 2017 and 2018.

Note: Standard errors clustered at the woreda level and reported in parentheses.
Statistical significance denoted by * p < 0.10, ** p < 0.05, *** p < 0.01.

Table E2: Health Extension Worker (HEW) characteristics by remoteness

|  | (1) | (2) | (3) | (4) | (5) | (6) |
| --- | --- | --- | --- | --- | --- | --- |
| *Outcome variable:* | *number of HEWs in the kebele* | *working hours per month* | *age in years* | *work experience (years)* | *education* | *test score* |
| Distance to woreda from kebele center (in minutes) | | | | | | |
| First tercile | (reference) | (reference) | (reference) | (reference) | (reference) | (reference) |
|  |  |  |  |  |  |  |
| Second tercile | -0.049 | 1.553 | -0.435 | -0.815 | -0.060 | -1.025 |
|  | (0.097) | (6.066) | (0.664) | (0.578) | (0.045) | (1.066) |
| Farthest tercile | 0.002 | 11.346 | -1.746*** | -2.516*** | -0.074 | -2.998** |
|  | (0.106) | (7.418) | (0.655) | (0.535) | (0.051) | (1.346) |
| Woreda fixed effects? | Yes | Yes | Yes | Yes | Yes | Yes |
| Observations | 249 | 248 | 249 | 249 | 249 | 249 |
| Within-R^2^ | 0.002 | 0.017 | 0.038 | 0.097 | 0.016 | 0.033 |

Source: Authors’ calculation based on the 2017 Productive Safety Net Program (PSNP) survey data.

Note: Standard errors clustered at the woreda level and reported in parentheses.
Statistical significance denoted by * p < 0.10, ** p < 0.05, *** p < 0.01.
